# Seeing redox at the microscale: 2D HERFD-XRF imaging of uranium oxidation states on thin sections of bacterial cells

**DOI:** 10.1039/d6ra04440a

**Published:** 2026-10-20

**Authors:** Stephan Hilpmann, Elena F. Bazarkina, Lucia Amidani, René Hübner, Kristina O. Kvashnina, Andrea Cherkouk

**Affiliations:** a Helmholtz-Zentrum Dresden-Rossendorf, Institute of Resource Ecology Dresden Germany a.cherkouk@hzdr.de +49 351 260 2989; b Rossendorf Beamline (BM20-ROBL), European Synchrotron Radiation Facility Grenoble France; c Helmholtz-Zentrum Dresden-Rossendorf, Institute of Ion Beam Physics and Materials Research Dresden Germany

## Abstract

Microbial U(vi) reduction plays a key role in the environment including potential deep geological repositories for high-level radioactive waste, as well as in bioremediation strategies for uranium-contaminated wastewater. HERFD-XRF mapping at the uranium M_4_ edge was applied to thin sections of U-reducing bacterial cells enabling oxidation-state-sensitive, spatially resolved detection of uranium.

## Abstract 2

The reduction of U(vi) to U(v) and U(iv) by bacteria can be followed by using HERFD-XRF and XANES measurements on thin sections, in combination with STEM-EDXS.

## Graphical abstract

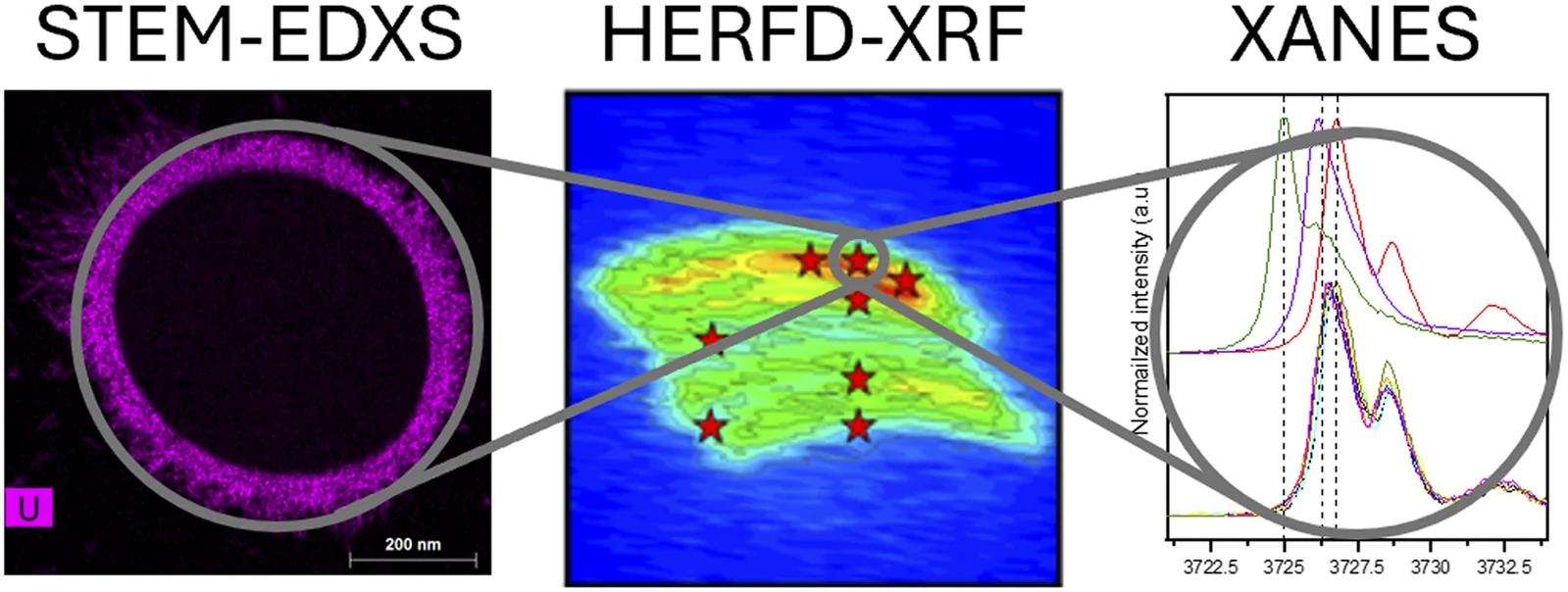

## Introduction

The microbial reduction of U plays a significant role in influencing the mobility and bioavailability of U in anoxic environments, including the engineered and natural barriers of potential deep geological repositories for high-level radioactive waste as well as uranium-contaminated sites.^1,2^ Sulfate- and metal-reducing bacteria can transform highly soluble U(vi) into U(v) and less mobile U(iv),^2–5^ which precipitates as uraninite^6–9^ or forms biominerals,^10^ thereby contributing to the long-term immobilization of radionuclides in the subsurface. However, the complexity of U redox chemistry, especially under environmentally relevant conditions, and the potential formation of metastable intermediate species such as U(v),^4,11,12^ demand advanced analytical techniques to study these processes at the microscale level.^13–15^

In recent studies, *Desulfosporosinus hippei* DSM 8344^T^, a sulfate-reducing bacterium, and *Desulfitobacterium* sp. G1-2, an iron-reducer, showed a reduction of U(vi) under anoxic conditions, resulting in the partial formation of U(iv). In the case of *D. hippei*, an unexpected stabilization of the transient intermediate U(v) was observed.^2,4^ These findings, based on bulk X-ray absorption spectroscopy (XAS), support the assumption that different bacteria may promote U valence states beyond the well-characterized U(iv) and U(vi). Such approaches provided crucial insights into average U oxidation state distribution. In combination with scanning transmission electron microscopy (STEM) coupled with element-specific energy-dispersive X-ray spectroscopy (EDXS) the cell ultrastructure and localization of U on and within the cell was demonstrated. However, the spatial co-localization of U oxidation states at the microscale is not possible with this technique. In particular, the oxidation state distribution of U across single cells and associated extracellular structures remained inaccessible.

To address this gap, we applied the non-destructive technique of high-energy-resolution fluorescence-detected X-ray fluorescence (HERFD-XRF) imaging^16^ at the U M_4_-edge to thin sections of U-reducing cultures of the bacteria *D. hippei* and *Desulfitobacterium* sp. G1-2. This represents the first reported application of U M_4_-edge HERFD-XRF mapping to thin sections of bacterial cells, providing spatially resolved and oxidation-state-sensitive detection of U.

The U M_4_-edge (3d_3/2_ → 5f_5/2_ transition) offers enhanced sensitivity to subtle differences in U oxidation states compared to the commonly used L_3_-edge, especially for distinguishing U(v) from U(iv) and U(vi),^4,17–19^ which is often difficult to achieve using conventional analytical or spectroscopic techniques.^13,14^ The combination of high-energy resolution and two-dimensional chemical imaging enables the detection of spatial heterogeneity in U oxidation states on the microscale in an area of several mm^2^. This advanced methodology was recently used to visualize the distribution of U(vi), U(v), and U(iv) oxidation in thin sections of different rock types.^16^ The approach opens new perspectives for investigating microbe-actinide interactions *in situ* and provides mechanistic insights into the microbial stabilization or transformation of redox-active radionuclides in complex environmental systems. In particular, high-energy-resolution fluorescence-detected X-ray absorption near-edge structure (HERFD-XANES) spectroscopy at the U M_4_-edge offers a powerful tool to identify and distinguish between complex mixtures of U(iv), U(v), and U(vi).^13,14,17,19–23^

In this study, we applied the above mentioned, non-destructive approach in actinide research by combining X-ray fluorescence (XRF) and HERFD techniques for thin sections of bacterial cells. The measurements were carried out using a Johann-type X-ray emission spectrometer^24^ at the BM20 (Rossendorf) beamline of the European Synchrotron Radiation Facility (ESRF).^25^ Additional details on the experimental setup have already been described elsewhere.^16,24^

The primary aim of this work was to evaluate the applicability of HERFD at the U M_4_ edge for spatially-resolved, two-dimensional mapping of U oxidation states in thin sections of bacterial cells containing U at ppm levels. Two types of bacterial cells from U(vi) reduction experiments were selected for analysis: (a) *Desulfosporosinus hippei* DSM 8344^T^, incubated for 48 h in artificial Opalinus Clay pore water^26^ with an initial U(vi) concentration of 500 µM,^4^ and (b) *Desulfitobacterium* sp. G1-2, incubated for 168 h in a 30 mM bicarbonate buffer with 100 µM of U(vi).^2^ Both bacterial strains showed U(vi) reduction within the respective incubation periods and differences in the localization of U in or on the cells.^2,4^

## Experimental

Samples for HERFD-XRF mapping of both bacterial strains were originally prepared for STEM measurements combined with EDXS. For this purpose, the cells were embedded in epoxy resin and cut in thin sections, which is a standard method for examining biological samples in TEM.^27,28^ The prior analyses with STEM-EDXS revealed U accumulation within^2^ and around^4^ the bacterial cells. For *D. hippei*, needle-like structures of a U compound were formed on the cell surface, whereas for *Desulfitobacterium* sp. G1-2 U nanoparticles were observed inside the cells. Detailed protocols for sample preparation can be found in the previous publications.^2,4^ For HERFD-XRF measurements, semi-thin sections (∼300 nm) were freshly cut from the existing epoxy resin blocks using a Leica UC6 ultra microtome and collected on carbon-coated slot grids. HERFD-XRF mapping was employed to these thin sectioned samples to demonstrate the feasibility of this method for sensitive, biological materials.

HERFD-XRF is an advanced variant of conventional XRF that provides detailed insights into the electronic structure of elements and improves spectral resolution (∼1 eV) by collecting fluorescence within a narrow energy window using a crystal analyzer, thereby suppressing background and enabling oxidation-state-sensitive mapping at concentration levels ranging from w% to ppm. Consequently, HERFD-XRF is particularly well suited for studying diluted or biologically relevant samples where target elements are present only in trace amounts. Moreover, HERFD-XRF complements element mapping by offering a powerful tool to probe the chemical and electronic environment of specific elements.

In this study, we applied HERFD-XRF at the uranium M_4_ absorption edge (3d_3/2_ → 5f transition), monitoring the U M_β_ (4f_5/2_ → 3d_3/2_ transition) emission line to obtain high-resolution spectral information. HERFD-XRF measurements were performed at the energies corresponding to the maxima of the U(iv) (3725.0 eV), U(v) (3726.0 eV), and U(vi) (3726.6 eV) oxidation states, as determined from reference spectra. By recording the fluorescence intensity (in arbitrary units) at these specific energies, we generated three distinct HERFD-XRF maps, each representing the spatial distribution of uranium in a particular oxidation state within the thin sections. These oxidation-state-resolved maps provide a valuable insight into the heterogeneity of uranium speciation and its transformation processes at the microscale. Evaluation of a potential X-ray-induced radiation damage was performed by making high speed scan measurements at the maximum of the emission line and at fixed incident energy around 3725 eV. No intensity variation was detected on samples upon scans over several minutes.

## Results & discussion

The HERFD-XRF maps obtained from the thin sections of *D. hippei* (Fig. 1a) confirmed the presence of all three U oxidation states (U(iv), U(v), and U(vi)), which is in good agreement with the previous study,^4^ where we showed that the intensities of U(v) and U(iv) are detectable but lower compared to U(vi). These previous bulk HERFD-XANES measurements of freshly prepared samples without epoxy embedding demonstrated that cells of *D. hippei* were capable of reducing up to 28% of U(vi) to mostly U(v) and U(iv) within 48 h.^4^ Despite the prolonged storage time of the samples, the distribution of the different U oxidation states remains clearly detectable. Although U(v) is generally considered unstable due to its tendency to disproportionate into U(iv) and U(vi),^29^ its persistence in the samples after TEM specimen preparation suggests remarkable stabilization, despite prolonged air exposure of the epoxy blocks. Another possible explanation for this stability could be a slow oxidation of U(iv) to U(v), resulting in a quasi-steady-state concentration of U(v) in the sample. However, as U(v) typically disproportionates faster than U(iv) oxidizes, this mechanism alone is unlikely to explain the observed persistence of U(v). Recent studies have shown that U(v) can be stabilized in iron-bearing mineral phases.^19,30–34^ Additional STEM-EDXS investigations of the thin sections revealed a co-localization of U and Fe on or within the bacterial cells (see Fig. 2), suggesting that biogenic iron-containing phases may contribute to U(v) stabilization in these systems.

**Fig. 1:**
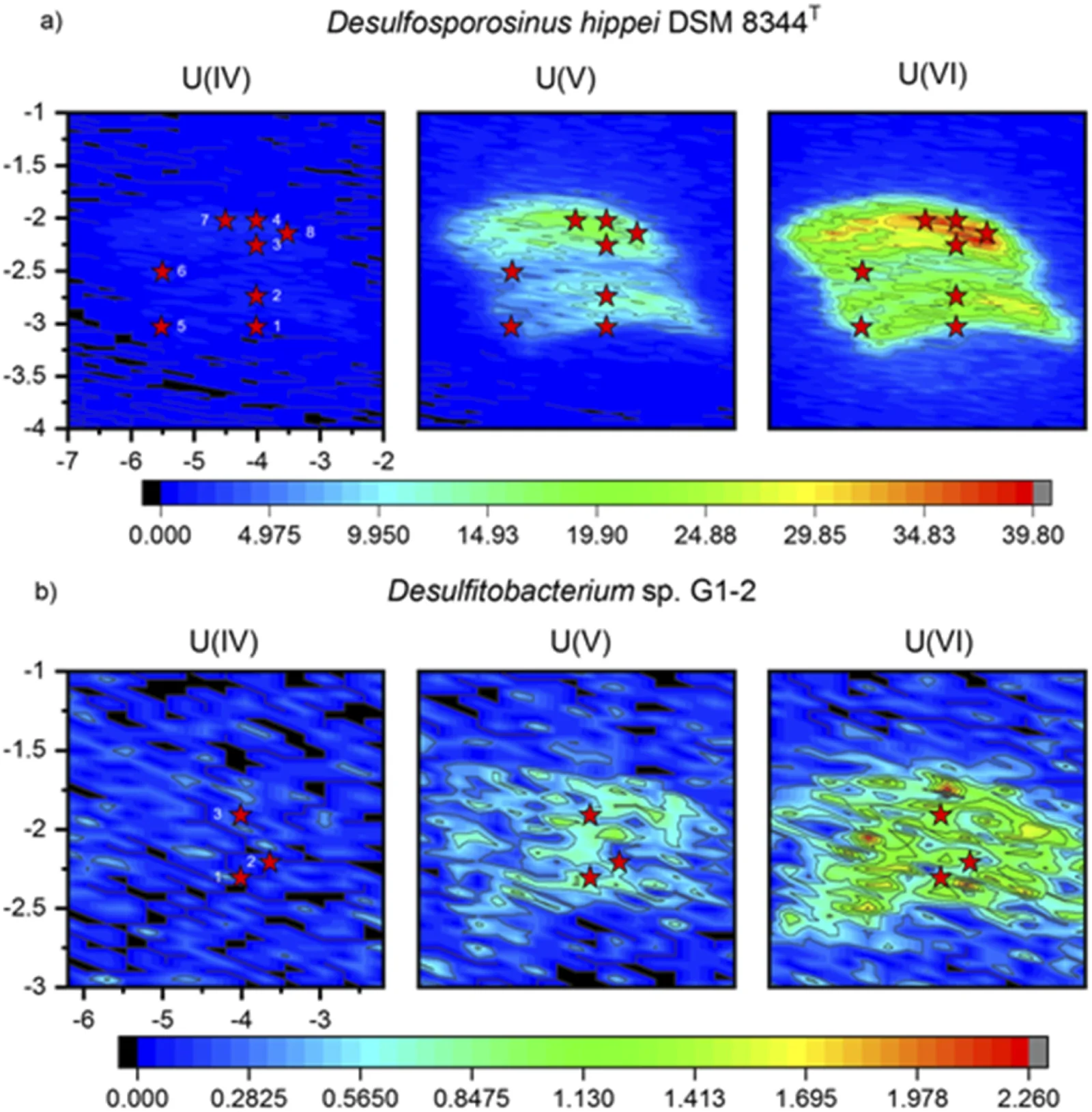
HERFD-XRF maps showing the distribution of U(iv), U(v), and U(vi) in thin sections (∼300 nm thickness) of (a) *Desulfosporosinus hippei* DSM 8344^T^ ([*U*_initial_] = 500 µM, 48 h incubation time, selected area of 3.0 mm (vertical) × 5.0 mm (horizontal)) and (b) *Desulfitobacterium* sp. G1-2 ([*U*_initial_] = 100 µM, 168 h incubation time selected area of 2.0 mm (vertical) × 4.0 mm (horizontal)) after microbial U(vi) reduction.^2,4^ Red stars mark the points of the measured HERFD-XANES single spectra. Please note that the color scales for (a) and (b) do represent different intensities.

**Fig. 2:**
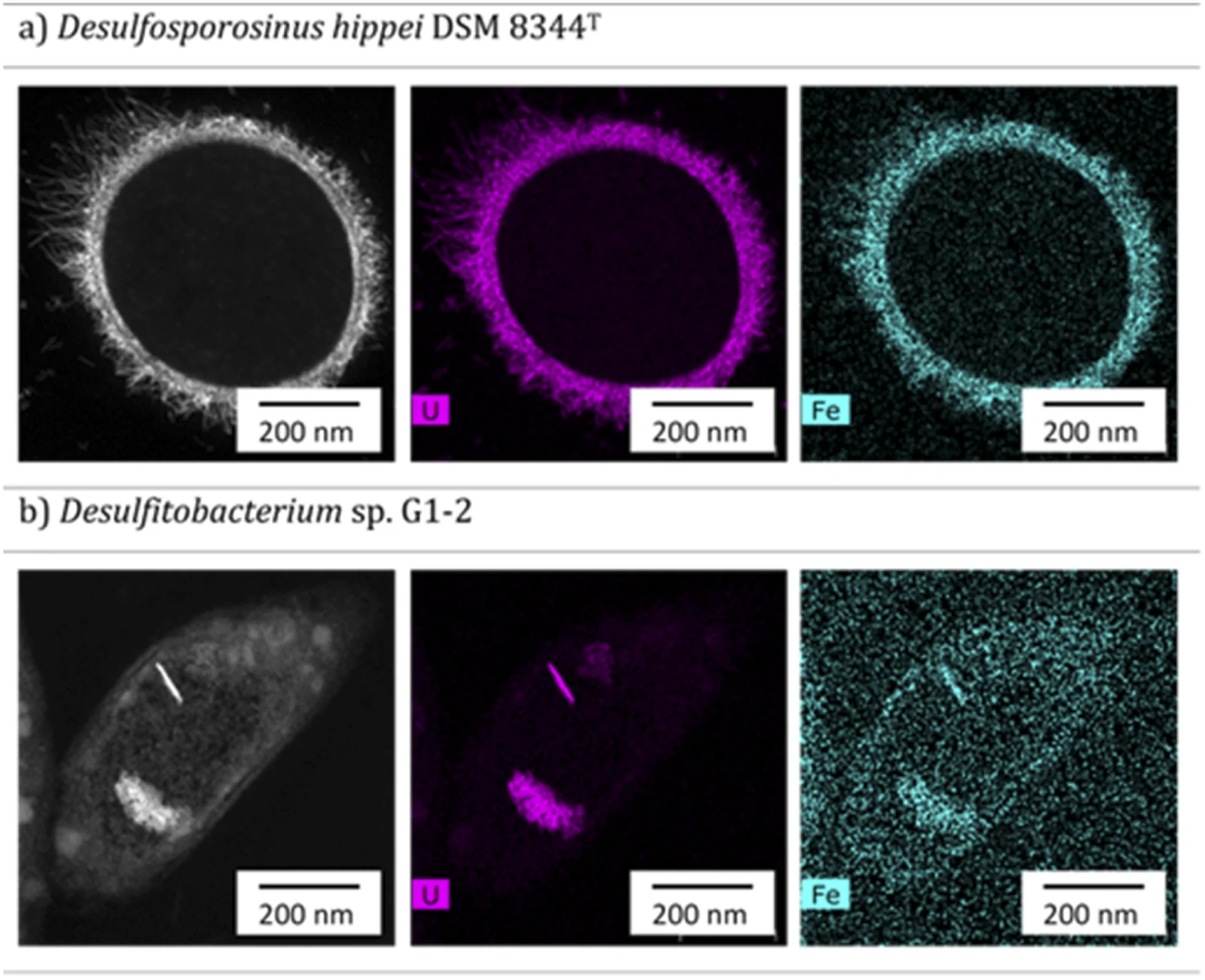
Representative HAADF-STEM images and corresponding U and Fe element distributions of ultrathin sectioned samples of (a) *Desulfosporosinus hippei* DSM8344^T^ cells treated with uranium ([*U*(vi)_initial_] = 500 µM) for 24 h and (b) *Desulfitobacterium* sp. G1-2 cells treated with uranium ([*U*(vi)_initial_] = 100 µM) for 48 h.

Notably, the maximum intensities of all U oxidation states are co-located in the same regions. This suggests that only the overall U concentration – and consequently the concentration of each oxidation state – varies across the thin section (Fig. 1). One potential reason for the lack of finer spatial resolution is the limited size of the incident X-ray beam (dimensions: 2 mm × 0.075 mm, which remains larger than the typical size of individual bacterial cells (∼1.0–1.2 µm for *D. hippei*)).^35^ However, HERFD-XRF image quality can be improved by oversampling, *i.e.*, using a step size smaller than the beam dimensions, which enhances spatial detail at the cost of longer acquisition times, as implemented in this study.^16^ Interpolation can additionally improve visual appearance without affecting the intrinsic resolution.

In contrast, the HERFD-XRF maps of *Desulfitobacterium* sp. G1-2 (Fig. 1b) display considerably lower overall signal intensities for each U oxidation state. This reflects the lower initial U(vi) concentration used in the bioreduction experiment, which was selected to explore the lower detection limit of this method. Despite this, a reduction of U(vi) in the samples was still measurable. Moreover, the detection of all oxidation states was possible, although the signal-to-noise ratio was notably lower. Considering the intensities of the various oxidation states, U(vi) again appears as the dominant oxidation state. Previous work has shown that *Desulfitobacterium* sp. G1-2 can achieve significant U(vi) reduction within one week.^2^ However, bulk HERFD-XANES studies have not yet been performed on those samples. With the help of HERFD-XRF mapping it was possible for the first time, to verify the presence of U(v) during U(vi) reduction by this bacterial species. The presence of U(v) in the current measurements indicates that this oxidation state may have been stabilized by the microorganism or its associated mineral phases, as well.

In summary, no consistent correlation between the spatial distributions of U oxidation states can be observed. While U(v) and U(vi) are broadly distributed across the sample, their intensity maxima in the maps do not overlap. In contrast, despite its low abundance, U(iv) has still been detected. This complex distribution may result from several factors, including the intracellular accumulation of U by *Desulfitobacterium* sp. G1-2, which could provide further localized protection from oxidation. This cellular uptake was already shown in our previous study.^2^ However, such cellular processes can currently not be resolved by the HERFD-XRF mapping.

It should be noted that the U(v) and U(vi) spectral features at the U M_4_-edge are located very close to each other, with only a small energy separation between their respective maxima. As a result, the HERFD-XRF maps recorded at the excitation energy corresponding to the U(v) state inevitably contain a minor contribution from U(vi) fluorescence. In contrast, the U(iv) signal is clearly distinguishable, as its spectral maximum is well separated from those of the higher oxidation states, making its distribution in the maps much easier to identify. Therefore, to unambiguously confirm the presence of pentavalent uranium, we performed line scans corresponding to HERFD-XANES spectra across representative regions of interest (8 points for *D. hippei* and 3 points for *Desulfitobacterium* sp. G1-2, indicated by red stars in Fig. 1; the points were selected to cover regions of the samples with different signal intensities). These spectra (see Fig. 3) were subjected to quantitative analysis using iterative transformation target factor analysis (ITFA),^33^ which enabled reliable identification and differentiation of the U(v) oxidation state from neighboring species and allowed the determination of the ratios of U oxidation states present in *D. hippei* and *Desulfitobacterium* sp. G1-2 thin sectioned samples. The number of principal components required to reproduce the spectra was determined in a first step using principal component analysis (PCA). Subsequently, the relative proportions of the three species corresponding to U(iv), U(v), and U(vi) were determined by the iterative target test (ITT) method implemented in the ITFA code. The HERFD method on single scans, in combination with ITFA, is a proven and reliable approach to quantify U(v) concentrations.^17,36,37^ This analysis indicates that for *D. hippei* 75–95% of U(vi), 1–30% of U(v), and 0–3% of U(iv) are present in different regions of the sample. In *Desulfitobacterium* sp. G1-2 sample, 79–87% of U(vi), 3–16% of U(v), and 6–12% of U(iv) were detected. For the analysis, UO_2_, UMoO_5_, and UO2^2+^ were used as pure U(iv), U(v), and U(vi) references, respectively, which have been proven to be good references for U oxidation state determination in biological samples.^4,38^ These distributions are consistent with the intensities of the HERFD-XRF maps measured independently.

**Fig. 3:**
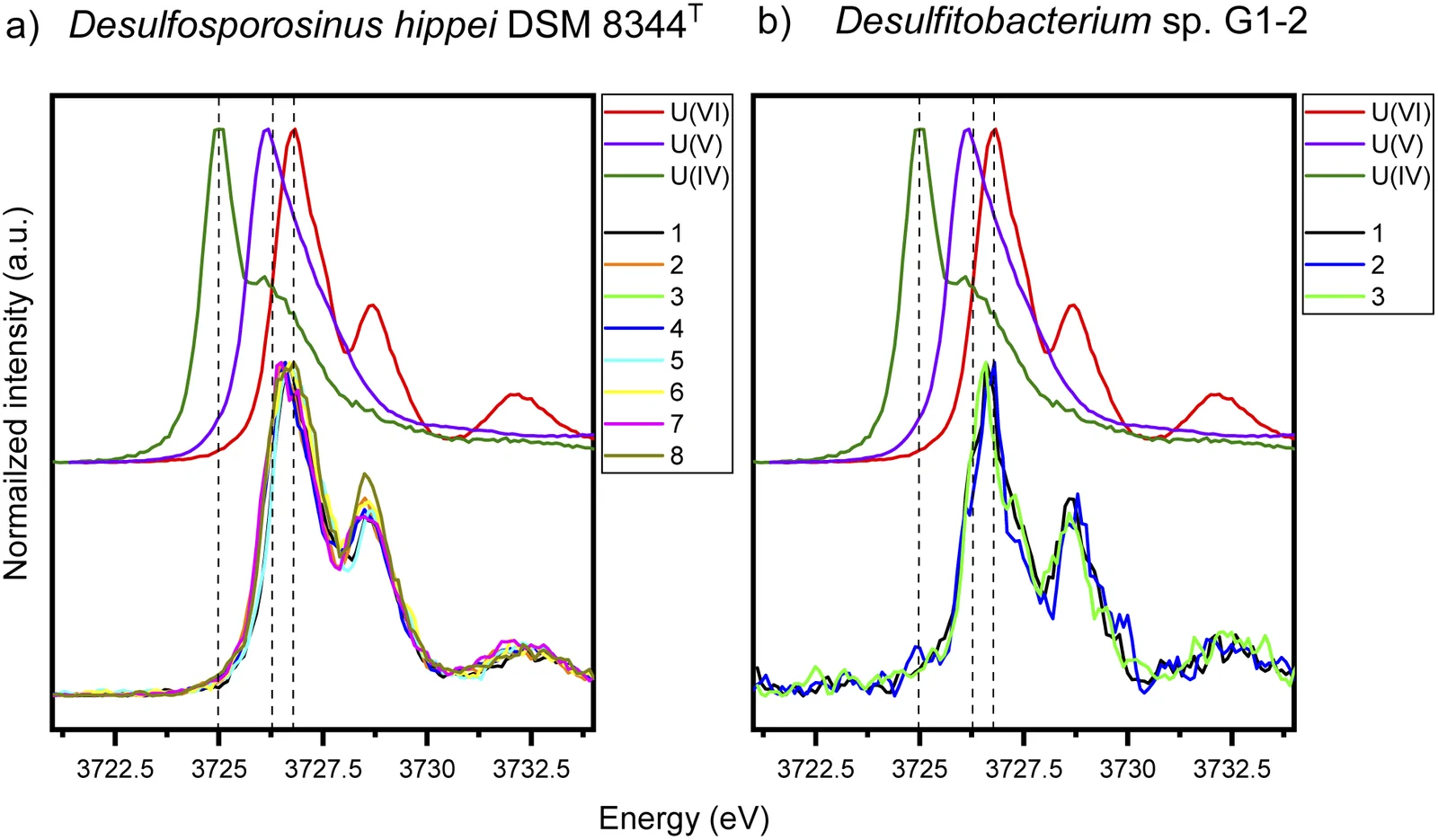
U M_4_-edge HERFD-XANES spectra measured at individual points (red stars in Fig. 1) in (a) *Desulfosporosinus hippei* DSM 8344^T^ and (b) *Desulfitobacterium* sp. G1-2 thin sectioned samples with the reference spectra of U(vi) as UO_2_^2+^, U(v) as UMoO_5_,^21^ and U(iv) as UO_2_.

A comparison of the proportions of the different U oxidation states between the two investigated microorganisms shows that *D. hippei* exhibits a higher overall proportion of U(v) in the spots than *Desulfitobacterium* sp. G1-2. This may be attributed to differences in the biogenic iron-bearing phases formed by *D. hippei*, which could more effectively stabilize the intermediate oxidation state, potentially due to structural variations within the formed reduction products between different strains and experimental conditions.^10,39^ Overall, the calculated U(vi), U(v), and U(iv) proportions are in good agreement with previous bulk HERFD-XANES measurements of *D. hippei*,^4^ supporting the remarkable stabilization of the reduced oxidation states in the samples.

In contrast, *Desulfitobacterium* sp. G1-2 shows a higher proportion of U(iv) at the measured spots, despite the overall low signal intensities observed in the U(iv) HERFD-XRF map. This finding suggests improved local protection of the reduced U, likely due to intracellular uptake of U during microbial reduction and is in good agreement with the higher initial proportion of U(vi) reduction by this microorganism.^2^ This shows that HERFD-XRF maps do not directly reflect single spot oxidation state proportions, as U(v) may dominate in the overall distribution while still coexisting with substantial amounts of U(iv) at single spots. To gain further insights into the structural nature of the U species, complementary extended X-ray absorption fine structure (EXAFS) spectroscopy would be valuable.

This first application of HERFD-XRF mapping to thin sections of bacterial cells serves as a proof of concept, demonstrating the viability of this highly sensitive technique for studying U oxidation state distributions in complex, biogenic matrices with very low concentration ranges up to ppm level. In addition, sample preparation time can be significantly reduced when thin sections originally prepared for TEM analysis to localize U on or within the cells can also be used for HERFD-XRF, which additionally provides information on U speciation. UV-Vis measurements of the bacterial cells prior to sample preparation should be performed to identify potential changes induced during preparation.^2,4^ In future work, reducing the X-ray beam size down to a few micrometers will significantly enhance the spatial resolution of HERFD-XRF measurements in order to accurately correlate oxidation state distributions with specific microstructural or biological features.

## Conclusions

HERFD-XRF imaging at the U M_4_-edge was shown to be a powerful tool for visualizing U oxidation state distribution in thin sections of bacterial cells. Significant amounts of U(v) were identified and quantified for both bacterial strains, indicating that biogenic U(v) phases, potentially in association with iron-bearing compounds, can stabilize this intermediate oxidation state for extended periods, and possibly longer. Furthermore, the presence of U(v) during U(vi) reduction by *Desulfitobacterium* sp. G1-2 is verified for the first time. Given the challenges in detecting U(v), it is likely underreported in many studies, and its role in environmental and engineered systems may be more significant than previously assumed. Furthermore, differences in U reoxidation behavior between the two microorganisms highlight differences in the localization of uranium within or on the cells as reported before. Ultimately, HERFD-XRF mapping offers broad applicability in actinide and environmental research.

## Author contributions

S. H., A. C. conceived the concept. S. H. performed the experiments and prepared the samples, S. H., A. C., E. F. B. performed measurements (2D HERFD-XRF imaging and HERFD-XANES spectra) at the Rossendorf beamline (BM20-ROBL) at ESRF, Grenoble, France, S. H., L. A., K. O. K. performed data analysis and created the figures. R. H. performed the STEM-EDXS analyses. All authors were involved in discussing experimental data, writing, and revising this manuscript.

## Conflicts of interest

There are no conflicts to declare.

## Data Availability

The datasets generated during and/or analyzed during the current study will be available in the Rossendorf Data Repository (RODARE) repository, (https://doi.org/10.14278/rodare.4995) and under Bazarkina, E., Cherkouk, A., & Hilpmann, S. Distribution of different uranium oxidation states in thin sections of uranium-incubated bacterial cells [Dataset]. European Synchrotron Radiation Facility https://doi.org/10.15151/ESRF-ES-2007905452.
